# Antitumor effects of lactate transport inhibition on esophageal adenocarcinoma cells

**DOI:** 10.1007/s13105-022-00931-3

**Published:** 2022-11-07

**Authors:** Laura Grasa, Eduardo Chueca, Samantha Arechavaleta, María Asunción García-González, María Ángeles Sáenz, Alberto Valero, Carlos Hördnler, Ángel Lanas, Elena Piazuelo

**Affiliations:** 1 IIS Aragón, Instituto de Investigación Sanitaria Aragón, Avenida San Juan Bosco 13, 50009 Saragossa, Spain; 2 Faculty of Veterinary Medicine, University of Zaragoza, Calle Miguel Servet, 177, 50013 Saragossa, Spain; 3 CIBERehd, Instituto de Salud Carlos III, Calle Monforte de Lemos 3-5, 28029 Madrid, Spain; 4 IACS Aragón, Instituto Aragonés de Ciencias de La Salud, Avenida San Juan Bosco 13, 50009 Saragossa, Spain; 5 Faculty of Medicine, University of Zaragoza, Calle de Pedro Cerbuna, 12, 50009 Saragossa, Spain; 6 Servicio de Patología, Hospital Universitario Miguel Servet, Paseo Isabel La Católica 1-3, 50009 Saragossa, Spain

**Keywords:** Esophageal adenocarcinoma, MCT, Lactate, Intracellular pH, Apoptosis, Proliferation

## Abstract

As a consequence of altered glucose metabolism, cancer cell intake is increased, producing large amounts of lactate which is pumped out the cytosol by monocarboxylate transporters (MCTs). MCT 1 and MCT4 are frequently overexpressed in tumors, and recently, MCT inhibition has been reported to exert antineoplastic effects. In the present study, MCT1 and MCT4 levels were assessed in esophageal adenocarcinoma (EAC) cells and the effects of the MCT-1 selective inhibitor AZD3965, hypoxia, and a glucose overload were evaluated in vitro. Two EAC cell lines (OE33 and OACM5.1C) were treated with AZD3965 (10–100 nM) under different conditions (normoxia/hypoxia) and also different glucose concentrations, and parameters of cytotoxicity, oxidative stress, intracellular pH (pHi), and lactate levels were evaluated. MCT1 was present in both cell lines whereas MCT4 was expressed in OE33 cells and only in a small proportion of OACM5.1C cells. Glucose addition did not have any effect on apoptosis nor cell proliferation. AZD3965 increased apoptosis and reduced proliferation of OACM5.1C cells, effects which were abrogated when cells were growing in hypoxia. MCT1 inhibition increased intracellular lactate levels in all the cells evaluated, but this increase was higher in cells expressing only MCT1 and did not affect oxidative stress. AZD3965 induced a decrease in pHi of cells displaying low levels of MCT4 and also increased the sodium/hydrogen exchanger 1 (NHE-1) expression on these cells. These data provide in vitro evidence supporting the potential of MCT inhibitors as novel antineoplastic drugs for EAC and highlight the importance of achieving a complete MCT inhibition.

## Introduction

The incidence of esophageal adenocarcinoma (EAC) has been increasing dramatically in the last decades in western countries [7]. The need for research of novel therapeutic strategies for this disease arises from the high resistance of this tumor to radiotherapy and chemotherapy linked to the fact that EAC is frequently diagnosed at advanced stages, resulting in poor prognosis and a 5-year survival rate lesser than 20% despite the use of combined therapies [15].

Abnormal pH homeostasis, an important hallmark of cancer cells, is related to increased proliferation, metastasis, and resistance to chemotherapy, and the reversal of this aberrant pH gradient has been linked to decreased tumor growth and metastases [19, 29]. Intracellular alkalinization and extracellular acidification are an early and crucial event in the neoplastic transformation which arises as a consequence of the overexpression of proton transporters in response to the expression of certain oncogenes [11, 28]. Following intracellular alkalinization, tumor cells also display a shift in glucose metabolism from mitochondrial oxidative phosphorylation to aerobic glycolysis, a phenomenon called the Warburg effect, which generates large amounts of lactic acid and implicates an increased rate of glucose uptake due to the low efficiency of glycolysis in terms of ATP production. In this way, glucose consumption in cancer cells is not determined by cellular needs but by extracellular glucose levels and its intake is facilitated by the overexpression of GLUT transporters (mainly GLUT1 and GLUT3) [1].

This phenomenon is being exploited for diagnostic purposes with positron emission tomography but could also be applied with therapeutic fine, e.g., to increase the cytotoxicity of some anticancer drugs, promoting lactic acid production and acidification in the tumor microenvironment by increasing the concentration of glucose. In this sense, previous works have shown that parental administration of glucose decreases pH selectively in tumor xenografts in rats without affecting systemic pH [34]. Interestingly, this effect was observed in tumors from different origins and histological types and was maximum at the highest glucose level (30 mM) evaluated, levels which can be achieved in clinical practice after intravenous infusion [16, 34].

To overcome the excess of acid production, cancer cells extrude protons through different transmembrane transporters among which monocarboxylate transporters (MCTs) catalyze the net transport of lactate anion with a proton. The MCT family spans 14 members of which MCT1 and MCT4 overexpression have been reported in several hematological and solid tumors such as the lung, liver, colon, melanoma, glioblastoma, breast, and ovarian cancers [8, 24, 32]. The distribution pattern of these MCTs varies among tumor types and even within the same tumor. MCT1 is expressed in the well-oxygenated areas of the tumor, whereas MCT4 expression is upregulated in hypoxic areas through hypoxia-inducible factor 1α (HIF-1α), that is a major regulator of adaptation to hypoxic stress widely associated with cancer progression [8, 24, 27, 32].

The relevance of targeting lactic acid transport and intracellular pH as an anticancer strategy is based on previous studies pointing out a link between a rise in extracellular lactate and increased cell migration, angiogenesis, escape of immune-surveillance, and tumor aggressiveness [3, 9, 17, 33]. In addition, several studies over the last decade have shown that pharmacological inhibition of proton-coupled monocarboxylate transporters alone or in combination with other pH disruptors can exert important antineoplastic effects on different tumors, especially on those with higher metabolic demands [4, 20, 25]. Since MCT1 and MCT4 expression is increased in EAC compared to normal esophageal epithelium [14] and the effects of MCT1 blockade in esophageal adenocarcinoma have not been investigated yet, we conducted a study to analyze the expression of the lactate transporters MCT1 and MCT4 in human EAC biopsies and cell lines and evaluated whether pharmacological inhibition of the constitutive transporter MCT1 can exert antineoplastic effects on two different EAC cell lines. In this study, we also examined the role that hypoxia and overproduction of lactic acid induced by glucose overload could have on these effects and the cellular mechanisms involved. Specifically, we will study the effects of MCT1 inhibition on levels of intracellular reactive oxygen species (ROS), as lactate accumulation can disrupt glycolytic flux, and thus increase ROS production through enhanced mitochondrial respiration to maintain ATP homeostasis [5, 20].

## Materials and methods

### Cell lines and culture conditions

Two EAC cell lines were used in this study. OE33 cell line, established from an adenocarcinoma of the lower esophagus arising from Barrett’s dysplasia, and OACM5.1C cells, established from a lymph node metastasis derived from a primary adenocarcinoma of the distal esophagus, were both purchased from ECACC (Salisbury, UK). Cells were cultured in RPMI-1640 supplemented with 10% FBS and 1% antibiotics (10,000 U/mL penicillin and 10 mg/mL streptomycin). To induce an increase in lactic acid production and tumor cell acidification, cells were also grown under a glucose overload (30 mM).

### MCT1 and MCT4 staining in biopsy samples and cell lines

In this study, archived esophageal biopsy specimens from patients with esophageal adenocarcinoma were obtained from the Service of Pathology of Miguel Servet University Hospital (Zaragoza, Spain). Each specimen was labeled with a study code, which did not include patient identifiers. The study was approved by the Ethical Committee of Clinical Research of Aragón (CEICA). As we used archived paraffin-embedded tissue and clinical data anonymously, including samples from deceased patients; no consent was considered to be obtained. Thirty-three esophageal adenocarcinoma biopsies were collected from archival paraffin-embedded samples using strict endoscopic and histological criteria, selected and revised by two pathologists (A.V. and C.H).

Immunohistochemistry was performed in paraffin-embedded biopsies and cell lines. Immunohistochemical staining was performed in 2.5-µm sample sections, cut, deparaffinized, and rehydrated. Cells were collected, fixed with 4% PFA, and pelleted with 1% agarose. Biopsies and cell sample sections were subjected to heat epitope retrieval at pH 9 using the PT Link module (Dako, Madrid, Spain). The samples were then incubated with primary antibodies to MCT1 (sc-365501; Santa Cruz Biotechnology, Dallas, USA) at dilution 1/150 and MCT4 (sc-50329; Santa Cruz Biotechnology) at dilution 1/300 using an automatic staining system (Dako Autostainer Plus). In the control experiments, primary antibodies were omitted as a negative control. Images were obtained using LAS EZ software (Leica, Barcelona, Spain) with a Leica DM 2500 microscope. The intensity of staining in esophageal adenocarcinoma samples was graded in the following categories: negative, mild, moderate, and intense.

### qPCR analysis of transporters

The relative abundance of MCT1, MCT4, sodium/hydrogen exchanger 1 (NHE-1), carbonic anhydrase-IX (CA-IX), and vacuolar-ATPase (V-ATPase) transporters in OE33 and OACM5.1C cell lines was measured by quantitative real-time PCR. The total RNA from EAC cell lines was isolated using the RNeasy Mini Kit (Qiagen, Hilden, Germany) followed by cDNA synthesis using the qScript cDNA superMix kit (Quanta bio, Beverly, MA, USA) according to the supplier’s protocol. PCR was performed with 200 ng of cDNA using the StepOne Plus Real-Time PCR System (Life Technologies, Carlsbad, California, USA). Specific primers for human MCT-1, MCT-4, SLC9A1(NHE-1), CA-IX, and ATP6V1C1 (V-ATPase) expression were designed and GAPDH and HPRT1 were selected as housekeeping genes (Table 1).

**Table 1:** Primers used for quantification of transporters in OE33 and OACM5.1C cells by qPCR

Gene	GenBank accession number	Sense and antisense primers
MCT1	NM_001166496.1	TTTCTTTGCGGCTTCCGTTGTTGTACGGA TCAATTTACCCTTCAGCCCCATGG
MCT4	NM_001206952.1	TTTTGCTGCTGGGCAACTTCTTCTG TCACGTTGTCTCGAAGCATGGGTTT
SLC9A1 (NEH1)	NM_003047.4	ACCTGGTTCATCAACAAGTTCCG TTCACAGCCAACAGGTCTACCA
CAIX	NM_001216.2	CACTCCTGCCCTCTGACTTC TCTCATCTGCACAAGGAACG
ATP6V1C1	NM_001695.4	ATGACTGAGTTCTGGCTTATATC AGCTACTTTCTTAACCACTCC
HPRT1	NM_000194.2	CCGGCTCCGTTATGGC GGTCATAACCTGGTTCATCATCA
GAPDH	NM_001289746.1	GAAGGTCGGAGTCAACGGATTT ATGGGTGGAATCATATTGGAA

Each sample was run in triplicate, the threshold cycle (CT) was determined, and relative gene expression was expressed as follows: change in expression (fold) = 2^−∆ (∆Ct)^ where ∆Ct = Ct (target)-Ct (housekeeping), and ∆(∆Ct) = ∆Ct (treated)-∆Ct (control).

### Apoptosis assay

The effects of AZD3965 (a MCT-1 selective inhibitor, 10–100 nM) or vehicle alone (DMSO) in the presence or absence of glucose overload (30 mM) were assessed by flow cytometry using the FACSAria cytometer (BD, Madrid, Spain). OE33 and OACM5.1C cells were stained with Annexin V-FITC and propidium iodide (PI). Apoptotic cells were defined as Annexin V and Annexin V + PI positive cells. Cells were seeded in 25 cm^2^ cell culture flasks and cultured until reaching 40–50% confluence. Then, cells were treated with AZD3965, incubated for 48 h under normoxic or hypoxic (1% O_2_) conditions, and collected for apoptosis determination. The experiments were repeated four times.

### Proliferation assay

Cell proliferation was measured using the BrdU assay kit (Roche, Barcelona, Spain) according to the manufacturer’s manual. Briefly, OE33 and OACM5.1C cells were seeded in 96-well plates and the next day AZD3965 (10–100 nM) or vehicle alone (DMSO) with or without glucose overload (30 mM) was added. After an incubation period of 48 h under normoxic or hypoxic (1% O_2_) conditions, cells were labeled with BrdU for 4 h and the labeling signal was quantified by measuring the relative absorbance (A_450_-A_690_ nm) with a plate reader (Synergy HT, Biotek, Winooski, USA). Each assay was done in triplicate and the experiment was performed four times.

### Measurement of intracellular lactate levels

Intracellular lactate levels were evaluated using a quantitative assay kit (Sigma-Aldrich, Madrid, Spain) following the manufacturer’s instructions. Briefly, cells were treated with AZD3965 (10–100 nM) or the vehicle (DMSO) with or without glucose overload (30 mM) under normoxic or hypoxic (1% O_2_) conditions for 48 h. Cells were then collected and lysed using the lysis buffer included in the kit. Then, lysates were filtered with a 10-kDa MWCO spin filter (Sigma-Aldrich, Madrid, Spain) to remove lactate dehydrogenase. Absorbance was quantified with the Synergy HT plate reader at 570 nm. Each sample was evaluated in duplicate and the experiment was repeated at least three times. The results expressed as nmol lactate/mg protein were normalized according to the protein concentration in each sample, which was quantified in cell lysates employing the BCA1 kit (Sigma-Aldrich, Madrid, Spain).

### Evaluation of cytosolic pH

Intracellular pH (pHi) was evaluated in OE33 and OACM5.1C cells by confocal microscopy using the pH-sensitive fluorescent probe C-SNARF-1 AM (ThermoFisher) as previously described [27]. Cells were seeded in 35 × 10 mm culture dishes and incubated at 37 ºC/5% CO_2_ in normoxia or hypoxia (1% O_2_) for 48 h to achieve 50% confluence. Cells were then treated with AZD3965 (100 nM) or vehicle (DMSO) for 30 min, 1 or 2 h and further stained with 5 μM C-SNARF-1 AM in serum-free media for 20 min. pHi was determined in an LSM 880 confocal microscope (Zeiss, Madrid, Spain) by recording two fluorescence data (phase 1: 543 nm excitation–580 nm emission and phase 2: 543 nm excitation–640 nm emission) and calculating the ratio (Phase 2/Phase 1), following the nigericin calibration procedure.

### Evaluation of reactive oxygen species (ROS)

The analysis of ROS production was assessed in OE33 and OACM5.1C cells at different points after AZD3965 addition using a quantitative assay (Abcam) based on ROS-sensitive probe DCFDA. 2.5 × 10^4^ cells per well were seeded in 96-well plates, and the next day, DCFDA probe and AZD3965 (100 nM) were added and incubated at 37 ºC. Intracellular ROS levels were evaluated every 1 h during 5 h or after 24 h of treatment and quantified as relative fluorescence units (RFUs) with respect to control cells measured at 495/529 nm using the Synergy HT plate reader. To evaluate if the addition of the antioxidant *N*-acetylcysteine (NAC) was able to reduce ROS levels, we also included cells treated with 5 mM NAC for 45 min before the addition of AZD3965.

### Drugs

Selective MCT1 inhibitor AZD3965 was obtained from MedChemExpress (NJ, USA). Glucose, nigericin, *N*-acetylcysteine (NAC), RPMI-1640, and antibiotics were purchased from Sigma-Aldrich (Madrid, Spain). Fetal bovine serum (FBS) and C-SNARF-1 AM were both from ThermoFisher (Madrid, Spain). All compounds except glucose solution, which was dissolved in water, and NAC, which was dissolved in culture media, were dissolved in DMSO and made up with the media so that the final concentration of the vehicle was not > 0.04% (v/v).

### Statistical analysis

Data analysis was performed using GraphPad (GraphPad Software, LaJolla, USA). Data were expressed as mean ± SEM. The Kolmogorov–Smirnov test was used to check if the data followed a normal distribution. Differences between groups were analyzed by the Mann–Whitney *U* test or the Kruskal–Wallis test as appropriate and *p* < 0.05 was considered statistically significant.

## Results

### MCT1 and MCT4 expression in esophageal adenocarcinoma biopsies and cell lines

**Fig. 1:**
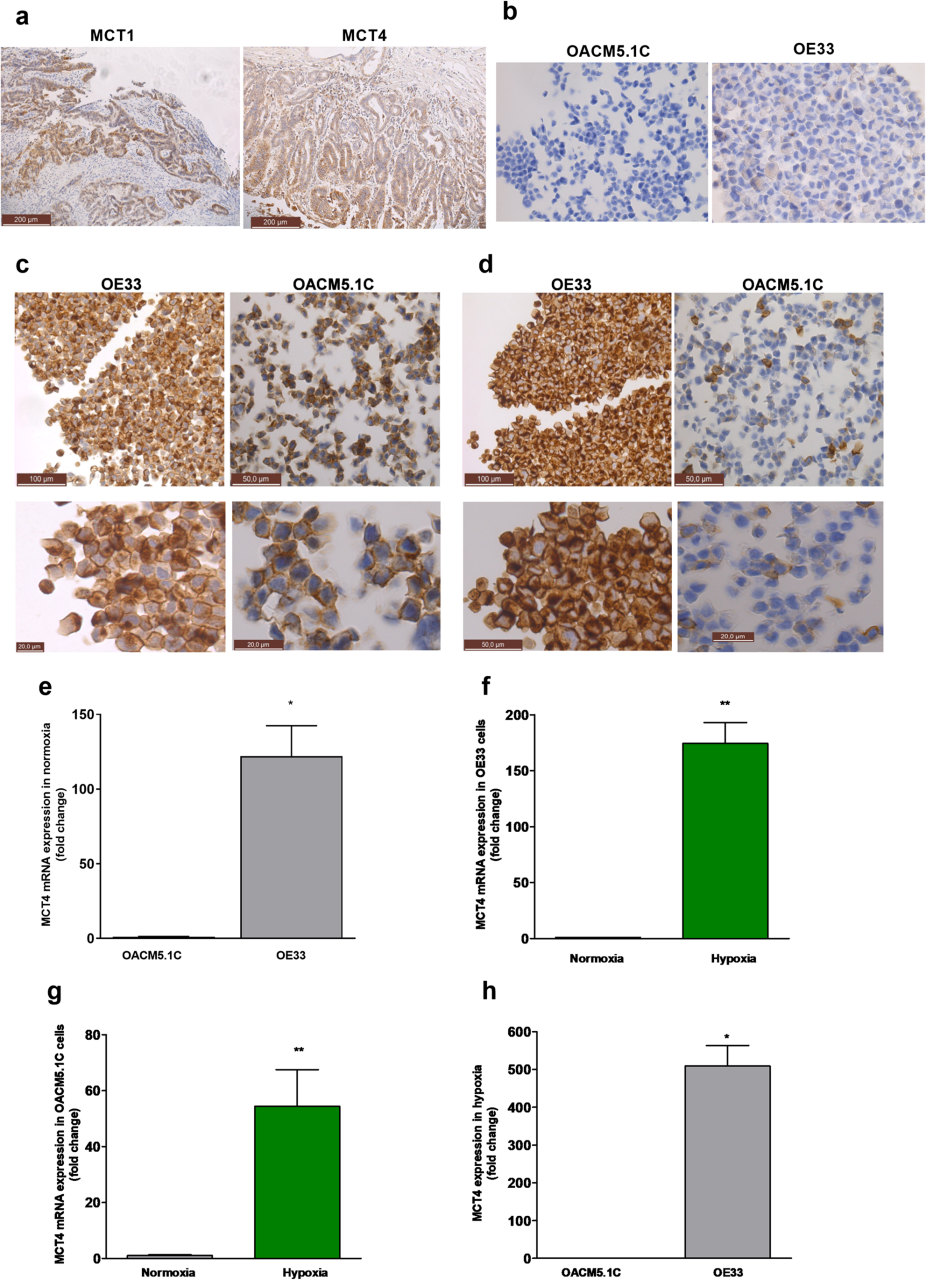
MCT1 and MCT4 expression in esophageal adenocarcinoma biopsies and OE33 and OACM5.1C cell lines. Immunohistochemical staining of MCT1 and MCT4 in esophageal adenocarcinoma biopsies (**A**). Negative controls of MCT1 in OACM5.1C and MCT4 in OE33 cell lines (**B**). Immunocytochemical labeling of MCT1 (**C**) and MCT4 (**D**) in OE33 and OACM5.1C cell lines. MCT4 relative expression was evaluated by qPCR comparing both cell lines in normoxia (**E**), comparing each cell line in normoxia vs hypoxia (OE33, **F**; OACM5.1C, **G**) and comparing both cell lines in hypoxia (**H**). All data are expressed as mean ± SEM of 4 independent experiments. Significant level **p < 0.01

MCT1 expression was found in all esophageal adenocarcinoma biopsies except one, with 45% of the biopsies showing moderate or intense staining. MCT4 staining was shown in all the tissue sections evaluated, with 75% of moderate/intense stained samples (Fig. 1A).

Immunocytochemistry revealed that both OE33 and OACM5.1C expressed MCT1 transporter (Fig. 1B, C), whereas MCT4 expression was present in OE33 cells and only in a small population of the metastatic OACM5.1C cell line (Fig. 1B, D). To confirm these results, we also quantified the relative expression of MCT4 in OE33 vs OACM5.1C in normoxia and the results showed that MCT4 expression in OE33 cells was significantly higher (122.09 ± 20.31 times, *p* < 0.01) than in the metastatic OACM5.1C cell line (Fig. 1E). Next, we evaluate whether hypoxia was able to increase MCT4 expression in our cell models, and the results showed an increase in MCT4 expression in hypoxia with respect to normoxia of 174.3 ± 18.8 (*p* < 0.01) times in OE33 and 54.41 ± 13.15 (*p* < 0.01) times in OACM5.1C (Fig. 1F, G, respectively). We finally compared the relative expression of MCT4 in OE33 vs OACM5.1C cells in hypoxia, and we observed again an important difference in the expression among both cell lines (508.78 ± 54.87 times higher in OE33 cells, *p* < 0.01) (Fig. 1, H).

### Effect of MCT1 inhibition on proton transporter expression

**Fig. 2:**
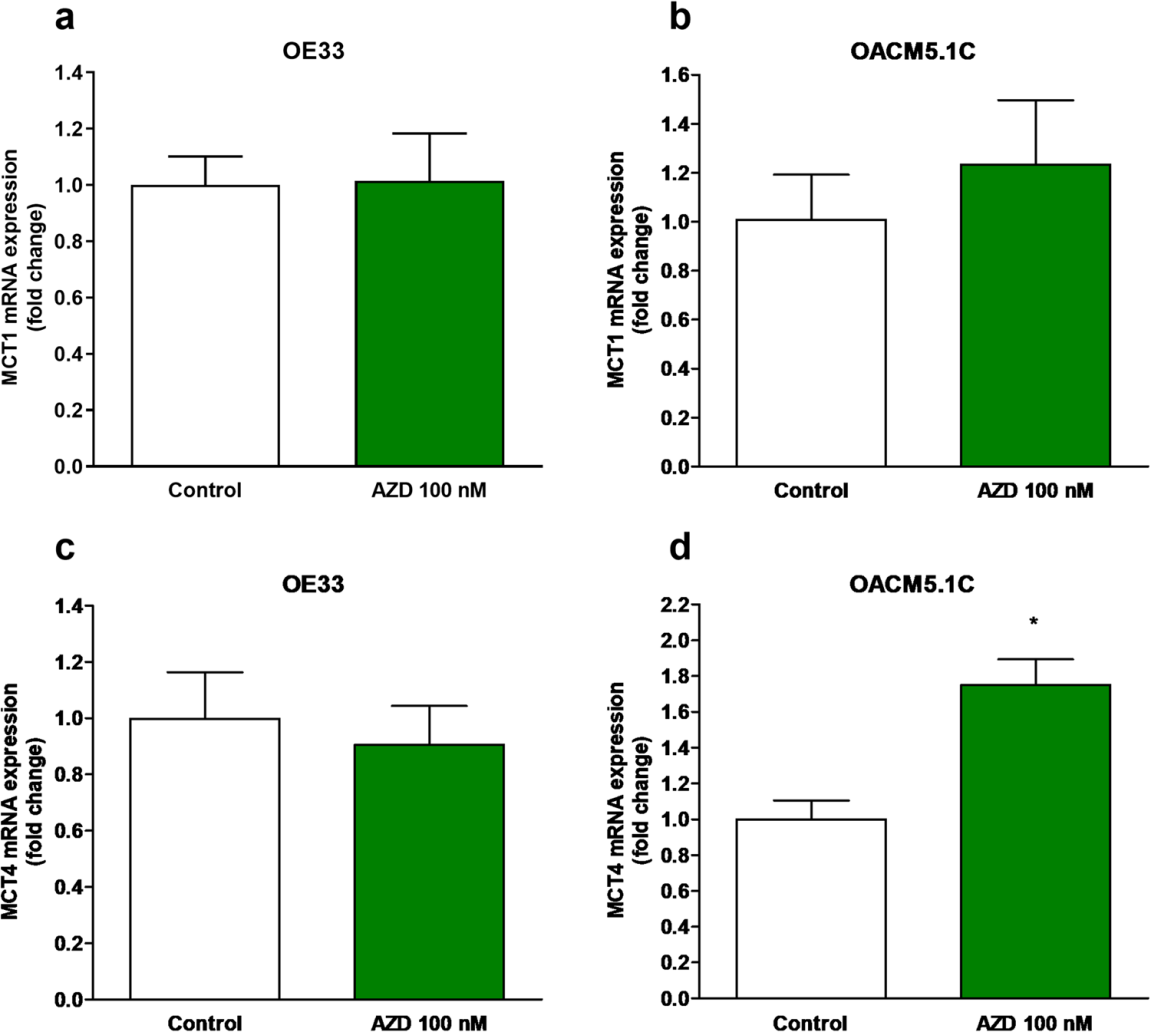
MCT1 and MCT4 relative mRNA expression in AZD3965-treated cells. MCT1 relative mRNA expression in OE33 (**A**), OACM5.1C cells (**B**), and MCT4 relative mRNA expression in OE33 (**C**) and OACM5.1C cells (**D**) was evaluated by qPCR after treatment with AZD3965 (100 nM) for 48 h, and the results are expressed as the level of expression of the transporters with respect to cells treated with the vehicle alone. All data are expressed as mean ± SEM of 4 independent experiments. Significant level *p < 0.05

**Fig. 3:**
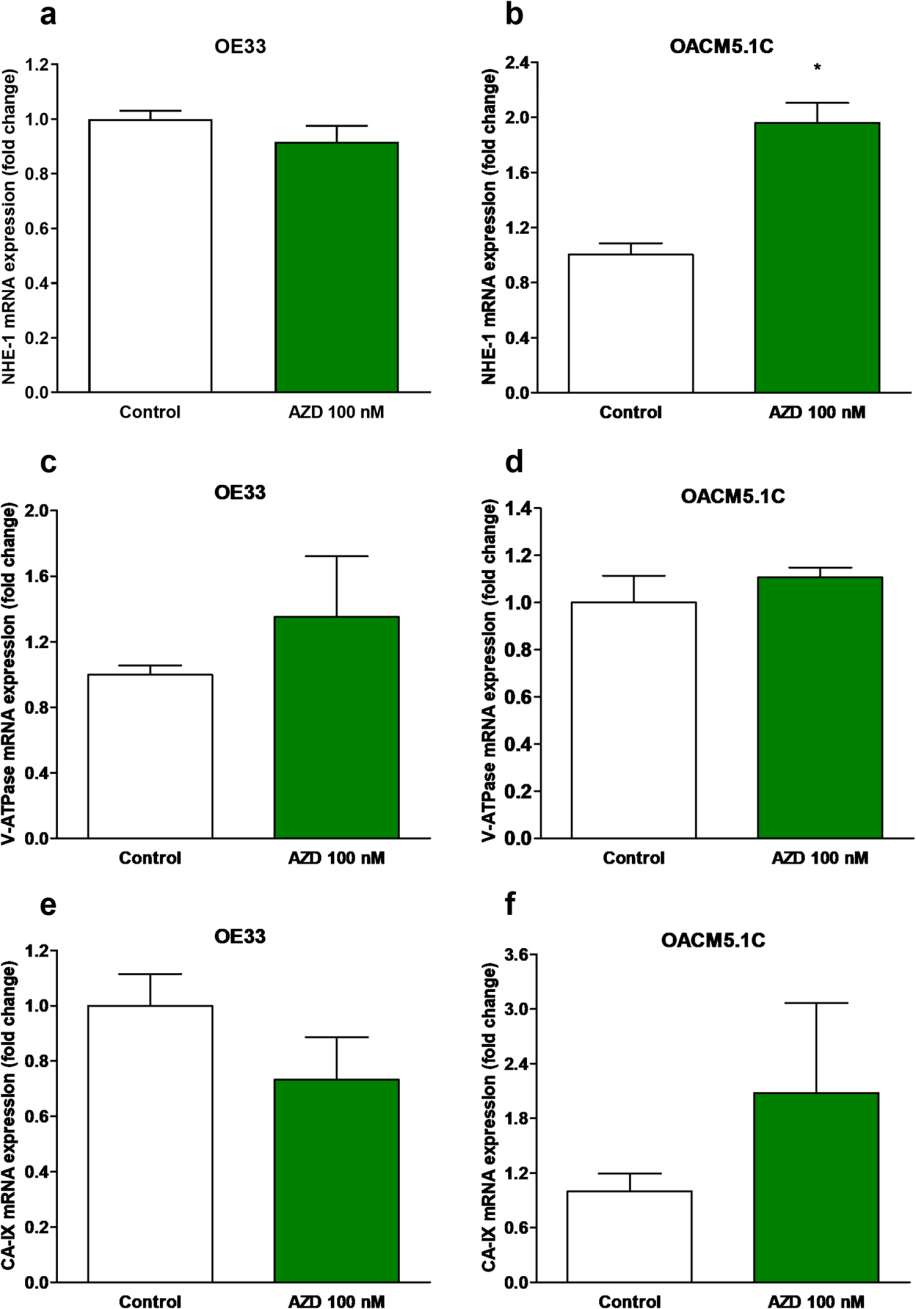
NHE-1, V-ATPase, and CA-IX relative mRNA expression in AZD3965-treated cells. NHE-1, V-ATPase, and CA-IX relative expression in OE33 (**A**, **C**, and **E**, respectively) and OACM5.1C cells (**B**, **D**, and **F**) was evaluated by qPCR after treatment with AZD3965 (100 nM) for 48 h, and the results are expressed as the level of expression of the transporters with respect to control cells treated with the vehicle alone. All data are expressed as mean ± SEM of 4 independent experiments. Significant level **p < 0.01

Since maintenance of intracellular pH is crucial for the survival of tumor cells [5, 6, 24, 28], we evaluate whether MCT1 inhibition was able to trigger overexpression of different proton transporters involved in pHi homeostasis. We then evaluated the effects of MCT1 inhibition on MCT1, MCT4, NHE-1, V-ATPase, and CA-IX expression by quantitative PCR. The results, expressed as the level of expression of the transporter in AZD3965-treated cells with respect to control cells (treated with vehicle alone), showed that treatment with AZD3965 for 48 h did not affect MCT1 expression on any of the cell lines, whereas an increase in MCT4 expression was found on the metastatic OACM5.1C cell line (Fig. 2D). Concerning other proton transporters, MCT1 inhibition did not affect NHE-1 expression (0.917 ± 0.06 vs 1.00 ± 0.03) in OE33 but increased its expression on OACM5.1C cells (1.96 ± 0.145 vs 1.00 ± 0.08, *p* < 0.01) (Fig. 3A, B). V-ATPase (Fig. 3C, D) and CA-IX (Fig. 3E, F) were not significantly affected by AZD3965 in any of the EAC cell lines evaluated.

### MCT1 inhibition induced apoptosis and reduced cell proliferation of metastatic OACM5.1C cells

**Fig. 4:**
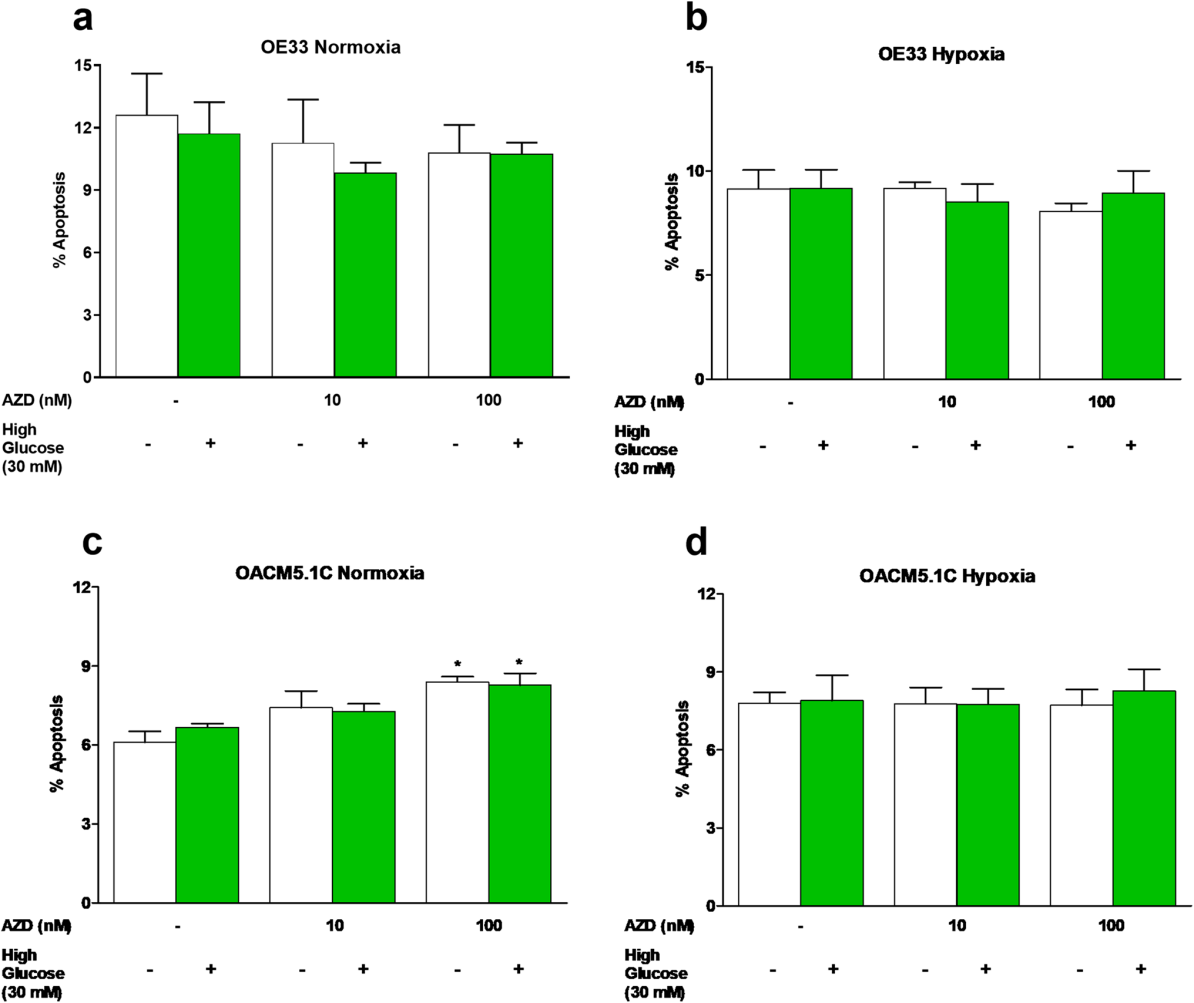
Effects of AZD3965 on apoptosis of EAC cell lines. Apoptosis was evaluated in OE33 (**A**, **B**) and OACM5.1C (**C**, **D**) cells in normoxia or hypoxia, respectively, in the presence or absence of a glucose overload (30 mM). The bars represent the mean % of apoptosis in AZD3965-treated cells with respect to control cells (DMSO only). All data are expressed as mean ± SEM of 4 independent experiments. Significant level *p < 0.05; **p < 0.01

**Fig. 5:**
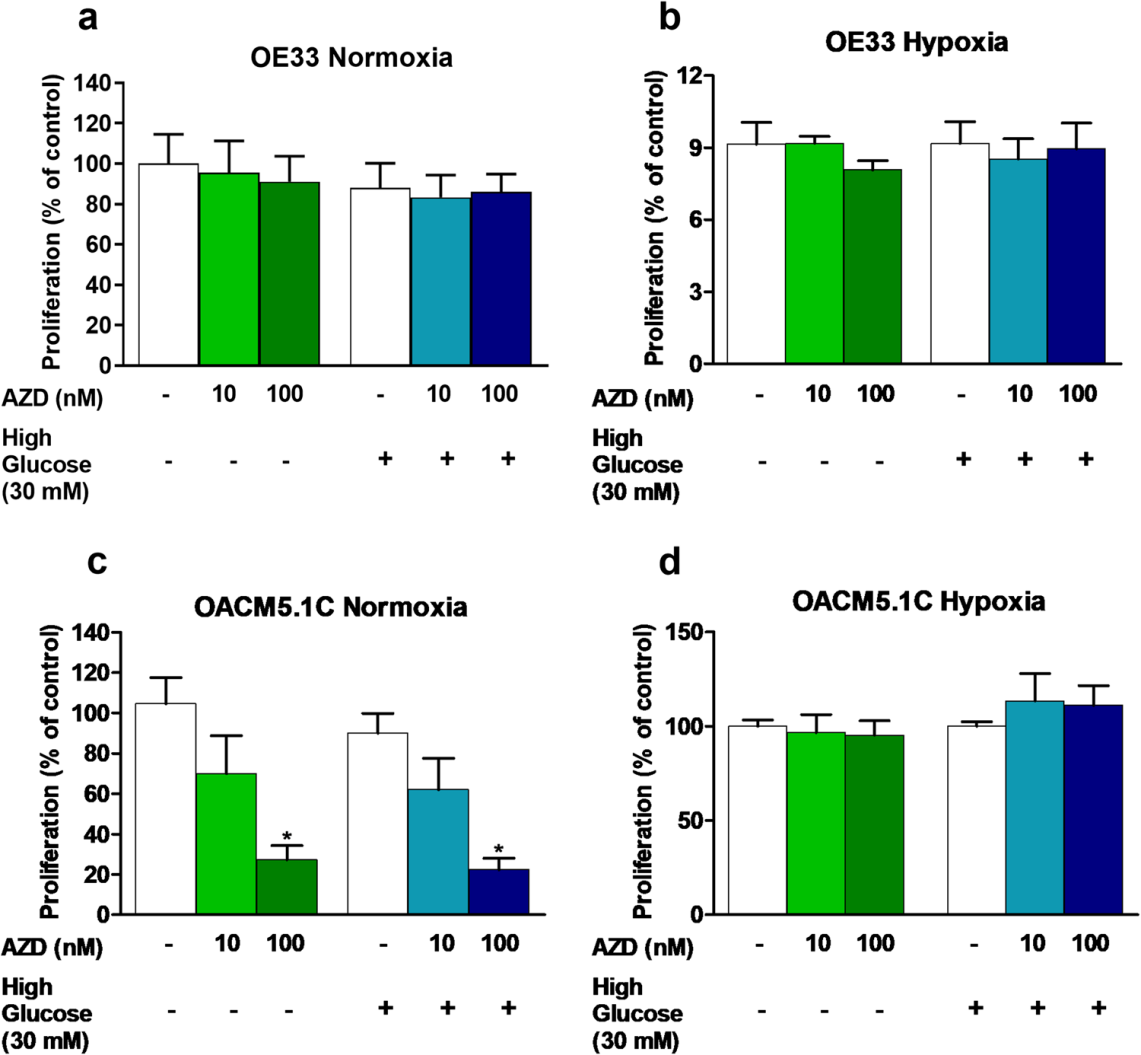
Effects of AZD3965 on cell proliferation. Cell proliferation in OE33 (**A**, **B**) and OACM5.1C (**C**, **D**) cells in normoxia or hypoxia in the presence or absence of a glucose overload (30 mM). The results are represented as the percentage of BrdU incorporation in AZD3965-treated cells in comparison with untreated controls (DMSO only). All data are expressed as mean ± SEM of 4 independent experiments. Significant level *p < 0.05; **p < 0.01

Given that glucose intake in cancer cells is increased at higher extracellular concentrations, we evaluated the proapoptotic effects of MCT1 inhibitor AZD3965 in the presence of standard (11 mM) and high (30 mM) glucose levels under normoxic and hypoxic conditions. MCT1 inhibitor did not have any effect on apoptosis of the non-metastatic OE33 cells under any of the conditions of normoxia or hypoxia evaluated (Fig. 4A, B), whereas slightly increased apoptosis of the metastatic OACM5.1C cell line only in normoxia (Fig. 4C). The addition of glucose did not affect the apoptosis of EAC cells. Since AZD3965 had different effects on apoptosis of EAC cells under normoxia and hypoxia, we next evaluate in the same conditions the effects of the inhibitor on cell proliferation in both cell lines. The results, expressed as % of BrdU incorporation in AZD3965-treated cells with respect to control cells, showed that the inhibitor did not affect the proliferation of OE33 cells (Fig. 5A, B), whereas significantly inhibited cell proliferation of the metastatic OACM5.1C cells in normoxia (Fig. 5C) but not under hypoxia (Fig. 5D). Glucose did not exert any effect on cell proliferation on any of the cell lines.

### MCT1 inhibitor increased intracellular lactate levels

We evaluated the effect of AZD3965 on intracellular lactate concentration in the presence or absence of a glucose overload (30 mM) under normoxic or hypoxic conditions.

**Fig. 6:**
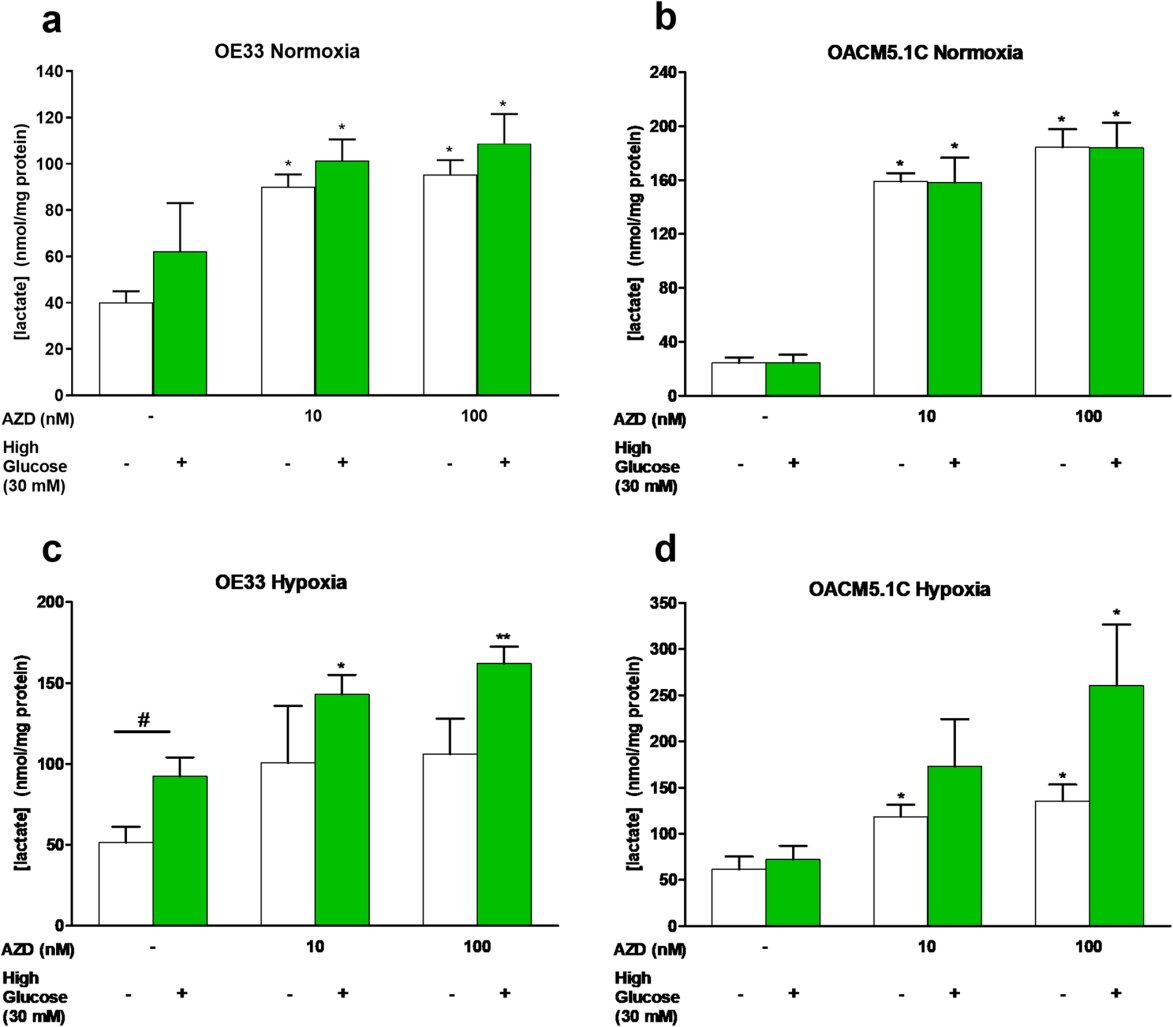
Effects of AZD3965 on intracellular lactate levels. Intracellular lactate levels in normoxia (**A**, **B**) or hypoxia (**C**, **D**) in the presence or absence of a glucose overload (30 mM). The results are expressed as nmol lactate/mg protein in AZD3965-treated cells in comparison with untreated controls (DMSO only). All data are expressed as mean ± SEM of at least 3 independent experiments. Significant differences from their respective control values: *p < 0.05; **p < 0.01. Significant differences from the respective treatment in the presence or absence of a glucose overload: #*p* < 0.05

MCT1 blockade significantly increased intracellular lactate levels in OE33 and OACM5.1C cell lines at 100 and 10 nM, both in normoxia (Fig. 6B) and hypoxia (Fig. 6C, D), but this increase was higher in the metastatic OACM5.1C cell line. OE33 cells in normoxia displayed similar intracellular lactate levels to those observed in hypoxia (Fig. 6A, C), whereas OACM5.1C cells grown in normoxia exhibited higher levels than hypoxic cells (Fig. 6B, D). Glucose overload had little effect on EAC cells in normoxia but induced a marked increase in intracellular lactate in hypoxia (Fig. 6C).

### MCT1 inhibition reduced the intracellular pH of metastatic cells

**Fig. 7:**
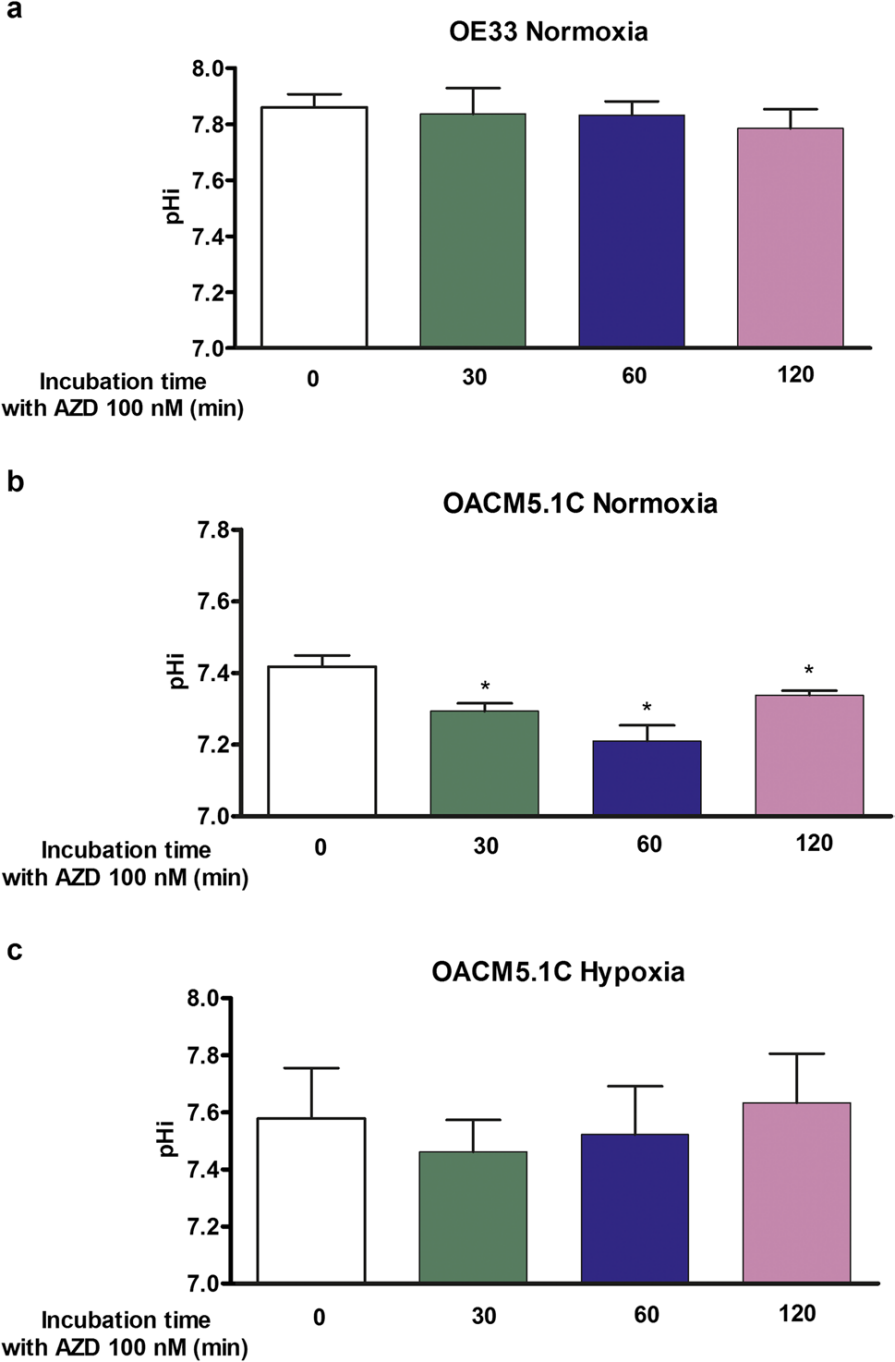
Effects of AZD3965 on intracellular pH. pHi was evaluated at 30, 60, and 120 min after AZD3965 (AZD) addition in OE33 cells in normoxia (**A**) and OACM5.1C cells in normoxia (**B**) or hypoxia (**C**). Data are represented as mean ± SEM of 3 independent experiments. Significant level *p < 0.05

We treated cells with AZD3965 (100 nM) for 30 min, 1 or 2 h to evaluate whether the MCT1 inhibitor was able to promptly induce changes in pHi in EAC cells. The results showed a basal alkaline intracellular pH in normoxia in OE33 cells (7.86 ± 0.048) that was not significantly affected by AZD3965 after 30 min (7.84 ± 0.093), 1 h (7.83 ± 0.049), or 2 h (7.78 ± 0.068) of treatment (Fig. 7A). Conversely, the metastatic OACM5.1C cell line displayed a basal intracellular pHi of 7.40 ± 0.023 in normoxia, and AZD3965 reduced pHi. After 30 min of treatment, there was a significant reduction in pHi (7.29 ± 0.022, *p* < 0.05), which was maximum after 1 h (7.21 ± 0.022, *p* < 0.05). After 2 h, pHi increased but remained reduced in comparison to control cells (7.34 ± 0.012, *p* < 0.05) (Fig. 7B). Since only the metastatic OACM5.1C cells were affected by AZD3965, we then evaluated whether MCT1 inhibition also affected pHi under hypoxic conditions in this cell line. The results showed that in hypoxia, basal pHi was 7.58 ± 0.18, and AZD did not significantly affect intracellular pH after 30 min (7.46 ± 0.11), 1 h (7.52 ± 0.17), nor 2 h (7.63 ± 0.17) (Fig. 7C).

### AZD3965 did not affect ROS levels in EAC cells

**Fig. 8:**
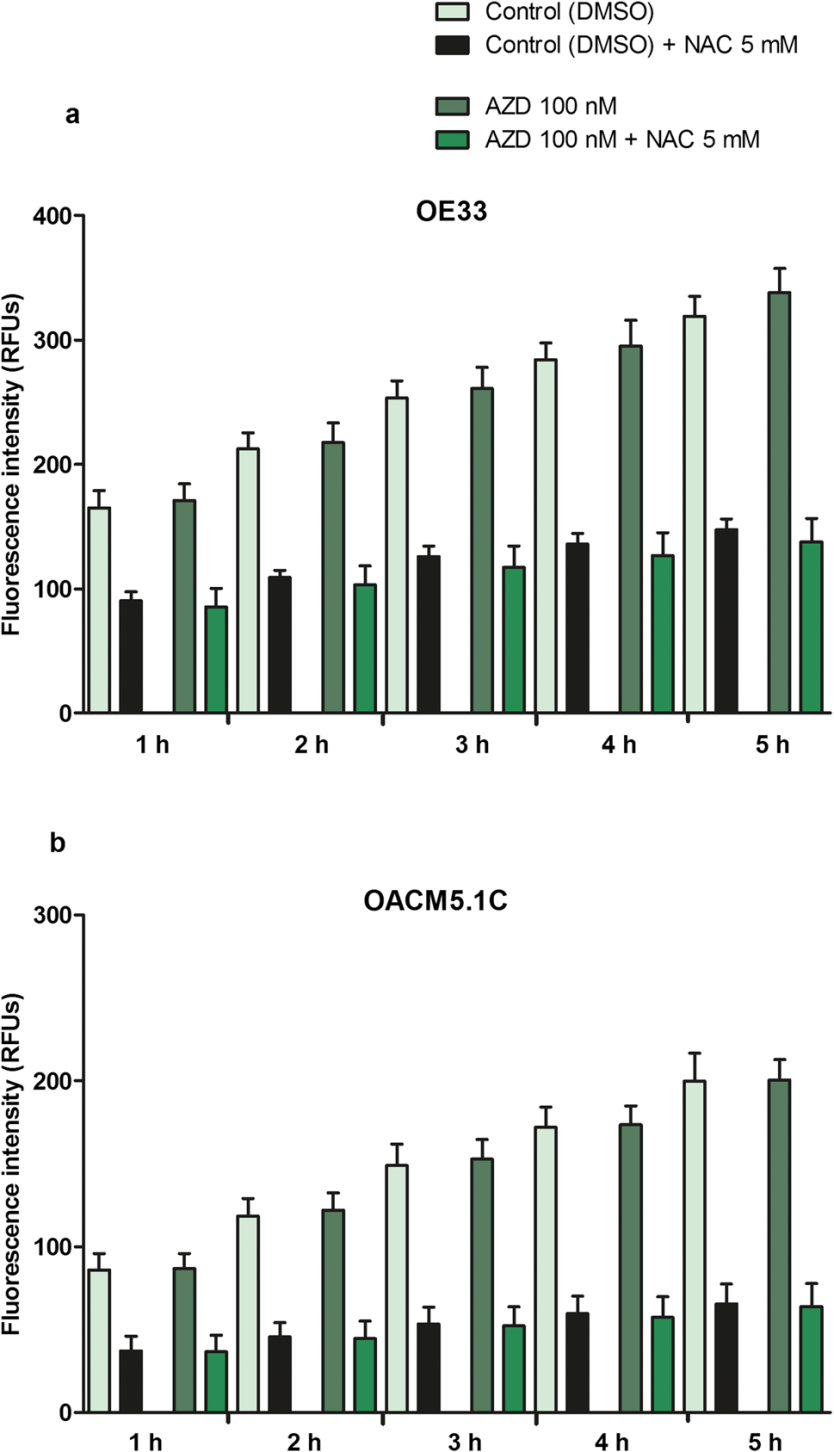
Effects of AZD3965 on ROS production in EAC cells. ROS levels in OE33 (**A**) and OACM5.1C cells (**B**) at different time points after the addition of AZD3965 (AZD) or AZD + the antioxidant NAC. All data are expressed as mean ± SEM of 3 independent experiments

We first studied whether there was a short-term increase in ROS production after adding AZD3965 to EAC cells. The results, expressed as RFUs, showed that MCT1 blockade did not affect ROS production on OE33 and OACM5.1C cells 5 h after adding the treatment (Fig. 8).

## Discussion

Despite its lower efficiency in ATP production in comparison to oxidative phosphorylation, glycolysis is a quick pathway to provide energy and metabolic precursors for fast-proliferating cells. Tumor cells rely on high glucose consumption and also on the ability to continuously pump out the large amounts of lactate and H^+^ generated by their metabolism to maintain the glycolytic process and to avoid intracellular acidification and metabolic inhibition caused by lactate accumulation. In this study, we attempted to force intracellular acidosis of esophageal tumor cells and, as a consequence, cell death, by increasing glucose concentration in the culture medium and simultaneously inhibiting the removal of H^+^ and lactate produced by using the selective MCT1 inhibitor AZD3965. Although treatment of cells with AZD3965 induced a significant decrease of pHi and cell proliferation and an increase of cell apoptosis only in the OACM5.1C cell line, higher glucose concentration in the culture medium did not have any effect on the cytotoxicity of the MCT1 inhibitor in any of the cell lines used. It is possible that our cell lines exhibit a phenotype that makes them insensitive to glucose overload as it has been previously described that multidrug-resistant cancer cells were unaffected by high glucose levels while drug-sensitive cells exhibited increased proliferation rates in response to glucose as a consequence of low PKC- βII expression [35].

Monocarboxylate influx and efflux is an active process carried out by MCTs, of which MCT1 and MCT4 have been shown to play a key role in maintaining an alkaline pHi in tumors [16]. Lactate is the main substrate for MCTs, and in vitro and in vivo studies have demonstrated that a blockade of lactate export and the subsequent pHi disruption is an effective strategy to target highly glycolytic tumor cells [4, 21, 23, 25, 31]. In this context, the pharmacological MCT1 inhibitor AZD3965 is currently undergoing a phase I study in patients with advanced cancer (NCT01791595).

Our data demonstrate that sensitivity to the cytotoxic effects of MCT1 inhibition varies among cell lines and also depends on oxygen levels. In normoxia, metastatic OACM5.1C cells were affected by MCT1 inhibition whereas the non-metastatic OE33 cell line remained unaffected, and both cell lines were insensitive to AZD3965 under hypoxia. Previous works showed that co-expression of both MCT1 and MCT4 transporters was related to resistance to MCT1 inhibition, which is consistent with the functional redundancy of MCTs [18, 21, 25]. Moreover, MCT4 knockdown or silencing in different highly aggressive cancer models in vivo and in vitro has been reported to make them sensitive to MCT1 inhibition [18, 20]. In our study, immunocytochemistry showed that metastatic OACM5.1C cells in normoxia expressed high levels of MCT1, with only a small proportion of the cells expressing MCT4, while OE33 cells expressed both MCT1 and MCT4 and remained unaffected by AZD3965 treatment. According to these results, we next sought to evaluate whether incubation under hypoxic conditions was able to increase MCT4 expression in our cell lines, as previous studies demonstrated that hypoxia induces MCT4 expression through HIF1- α [33]. In line with these studies, both OE33 and OACM5.1C cells showed an increase in MCT4 expression after 48 h growing in hypoxia, and thus, AZD3965 effects on the metastatic OACM5.1C cell line were completely abolished, suggesting that complete inhibition of MCTs is required to exert antineoplastic effects. Thus, empirical results reported in the present study should be considered in light of the limitation of the lack of simultaneous pharmacological inhibition of both MCTs, since to date there are no commercially available MCT4 selective inhibitors.

In this work, we decided to evaluate apoptosis and proliferation after 48 h of treatment based on previous studies with our cell model in which we observed only a slight effect in apoptosis when apoptotic inducers were used for less than 48 h.

Our results showed that MCT1 inhibition in normoxia induced growth arrest and had little effect on apoptosis on AZD3965 sensitive cells, effects which could be induced at least partially by the intracellular acidification observed in these cells. To further elucidate the cellular mechanisms involved in the observed cytotoxic effects, in the present work, we also evaluated pHi and lactate after AZD3965 addition. Under normoxic conditions, MCT1 inhibition decreased pHi of OACM5.1C cells but not OE33 cells and raised intracellular lactate levels in both EAC cell lines. However, this increase in lactate was remarkably higher in the metastatic OACM5.1C cell line, which together with the effects on pHi seemed to be a consequence of the different levels of MCTs expression between the cell lines. It should be noted that intracellular acidification in OACM5.1C cells may have been softened by the increase in MCT4 and NHE-1 expression, a proton pump implicated in pHi regulation in several tumors [2, 5, 12] observed after AZD3965 addition. Previous studies showed that acidification triggers NHE-1 expression and this response allows cells to fight the lethal cell acidosis[30]. Since OE33 cells expressed both MCT1 and MCT4 transporters, there was no variation in pH levels and thus expression of NHE-1 remained unaffected after AZD3965 addition.

As previously seen, intracellular acidification affects a wide range of cellular processes like cell growth, through a blockade in G2/M entry and completion of the S phase due to mTORC1 inhibition in response to intracellular acidic stress [6, 21, 26, 29]. Although the magnitude of internal pH changes observed in the metastatic cells after treatment with AZD3965 was small, previous reports observed on melanoma cells showed that even a slight variation in intracellular pH seems to be able to induce cell growth arrest [22]. Intracellular acidification has also been linked to increased apoptosis in cancer cells, creating the optimal conditions for the activation of caspases and different apoptotic pathways [13]. In this study, MCT1 inhibition increased apoptosis in OACM5.1C cells, but these effects may have been lowered as a consequence of the small drop observed in intracellular pH.

In line with these results, it is feasible that we did not observe effects on intracellular pH, apoptosis, or cell proliferation upon MCT1 blockade in both cell lines growing under hypoxia as a consequence of MCT4 increased expression. Results also showed that in these conditions, lactate increased to a lesser extent than in normoxia.

Finally, we evaluated the effects of MCT1 inhibition on intracellular ROS levels. High intracellular lactate levels have been previously linked with cytotoxic effects as a consequence of an increase in oxidative stress. It has been proposed that lactate accumulation might disrupt glycolytic flux, and thus increase ROS production through enhanced mitochondrial respiration to maintain ATP homeostasis [5, 20]. This was not the case for OE33 and OACM5.1C cells, in which treatment with MCT1 inhibitor at different times did not affect ROS levels, a result consistent with the antioxidant character of lactate [10], suggesting that in our model AZD3965-induced cytotoxic effects might be independent of ROS generation.

The present work has shown that targeting MCTs is an effective strategy to inhibit tumor growth of EAC cells by inducing an increase in intracellular lactate levels and a decrease in intracellular pH when effective inhibition of lactate and protons efflux is achieved. In this sense, and considering the fact that within a tumor there is a continuous shuttling of lactate among highly glycolytic hypoxic tumor cells, pumping out lactate through MCT4, and oxidative tumor cells which can import and use the monocarboxylate as a source of energy, in addition to the cytotoxic effects that intracellular acidification exerts on tumor cells, the need for developing selective and potent proton and lactate transporters inhibitors arises as a valuable anticancer therapy.

## Conclusions

**Fig. 9:**
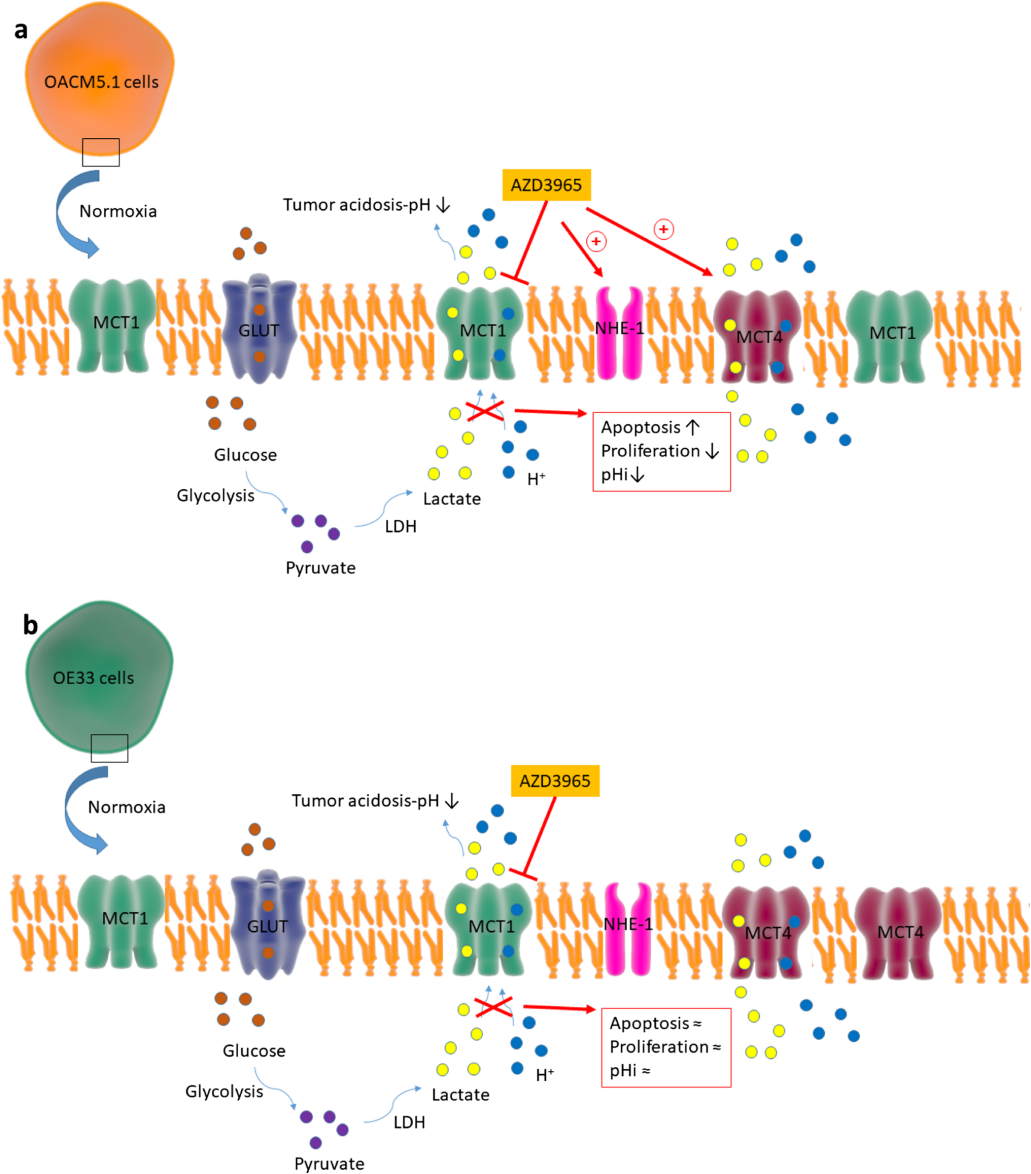
Graphical abstract of the effects of AZD3965 on OACM5.1C (**A**) and OE33 (**B**) cell lines. In OACM5.1C cells in normoxia, the inhibition of MCT1 by AZD3965 induced an increase in the expression of MCT4 and NHE-1, as well as an increase in apoptosis and a decrease in intracellular pH and proliferation (**A**). In OE33 cells in normoxia, AZD3965 did not induce significant changes in intracellular pH, proliferation, apoptosis, or in the expression of MCT4 or NHE-1 (**B**)

The main effects of the MCT1 inhibitor, AZD3965, on OACM5.1C and OE33 cells are shown in Fig. 9. Pharmacological inhibition of the MCT1 transporter exerted antineoplastic effects in EAC cells with low MCT4 expression, mainly by inhibiting proliferation, and in a lesser extent by inducing apoptosis. Increased extracellular glucose concentration did not modify the antitumor effect of MCT1 inhibition in these cell lines. Although MCT1 inhibition was associated to increased intracellular levels of lactate independently of MCT4 expression, cell acidification was only achieved when low levels of MCT4 transporter were expressed in EAC cells. Hypoxia was associated to an increase of MCT4 expression and abrogated the antitumor effect of MCT1 inhibition.

## Abbreviations

EAC: Esophageal adenocarcinoma
MCTs: Monocarboxylate transporters
pHi: Intracellular pH
HIF-1α: Hypoxia-inducible factor 1α
NAC: *N*-Acetylcysteine
FBS: Fetal bovine serum
NHE-1: Sodium/hydrogen exchanger 1
CA-IX: Carbonic anhydrase-IX
V-ATPase: Vacuolar-ATPase
ROS: Reactive oxygen species
